# “Getting stuck with LAM”: patients perspectives on living with Lymphangioleiomyomatosis

**DOI:** 10.1186/1477-7525-12-79

**Published:** 2014-05-21

**Authors:** Amanda Belkin, Karen Albright, Kaitlin Fier, Jennifer Desserich, Jeffrey J Swigris

**Affiliations:** 1 Autoimmune Lung Center and Interstitial Lung Disease Program, National Jewish Health, 1400 Jackson Street, Denver, CO 80206, USA; 2 Department of Community and Behavioral Health, Colorado School of Public Health and Colorado Health Outcomes Program, University of Colorado School of Medicine, Aurora, CO, USA; 3 University of Colorado School of Public Health, Aurora, CO, USA

## Abstract

**Background:**

Lymphangioleiomyomatosis (LAM) is a rare, progressive lung disease that affects almost exclusively women and is most often diagnosed before menopause. The main symptom of LAM is shortness of breath. LAM patients’ perceptions of how the disease impacts their lives is largely unknown, but such information could be useful to generate patient reported outcome measures for use in drug trials (or other research studies) and to formulate interventions aimed at easing the burdens LAM imposes on patients.

**Objective:**

To capture patients’ perceptions of how LAM affects their lives.

**Methods:**

We used reflexive team analysis to analyze transcripts from semi-structured focus groups conducted with LAM patients at LAMposium 2013. We sought to determine what patients perceive as the primary symptoms of LAM and how the disease affects them in their daily lives.

**Results:**

The 37 participants described seven primary symptoms of LAM and five common psychological experiences from living with the disease. Shortness of breath and low energy (or fatigue) dominated the symptomatic picture; cough, sensations in the chest, difficulty sleeping, gastrointestinal issues, and mild cognitive difficulties were less common. The common psychological experiences participants reported included frustration, worry, loss of identity, embarrassment, and in some participants, a healthy defiance against the disease.

**Conclusions:**

Patients perceive the physical symptoms from LAM to be intrusive and limiting. Women living with LAM are frustrated by their physical limitations, and they worry about what the future will be like if the disease progresses. Therapeutic interventions should take aim at improving these perceptions.

## Introduction

Lymphangioleiomyomatosis (LAM) is a rare, progressive and incurable lung disease, diagnosed predominantly in women of child-bearing age [1-3]. LAM occurs sporadically (S-LAM) or as a pulmonary manifestation of tuberous sclerosis complex (TSC-LAM) [4]. In either case, abnormal, smooth muscle-like cells (LAM cells), of unknown origin, infiltrate the lung and cause the formation of innumerable, thin-walled cysts. As functional lung parenchyma is replaced by space-occupying, air-filled cysts, patients become increasingly short of breath—in most cases, hypoxemia develops within ten years of symptom onset [3,5,6]. The predominant symptom of LAM is exertional dyspnea; cough is variably present; and pneumothorax may occur either as the initial manifestation, leading women to seek medical attention, or at other times throughout the course of the disease [7].

In a recent trial, sirolimus was found to halt LAM progression, offering women with LAM a viable treatment option and, for the first time, hope against the ravages of the disease [8]. Unfortunately, use of sirolimus is not without problems. It is not uniformly effective, is not tolerated by all women with LAM, and does not cure the disease. Further, it possesses a number of potential side-effects, requires routine blood monitoring, and questions remain about when to initiate therapy with sirolimus. Thus, despite the hope that sirolimus has brought to the entire LAM community, many women with LAM will continue to see their conditions worsen, may require lung transplantation and, ultimately, may die from disease progression.

Based on existing questionnaires, relative to the general population, women with LAM have impaired quality of life, particularly in domains that assess physical functioning [7]. However, to our knowledge, a qualitative study with the primary aim to capture patients’ perceptions of how LAM affects their lives, has never been conducted. This leaves a large gap in our understanding of the true impact of LAM on the individuals with this disease. We conducted this qualitative study to capture and carefully examine LAM patients’ perceptions of the burdens of this disease and to gain a clearer understanding of how the disease affects them in their daily lives.

## Methods

We used convenience sampling to recruit participants from the LAMposium meeting in Cincinnati, Ohio in April 2013. At that meeting, we conducted seven semi-structured focus groups (FGs) with LAM patients to investigate participants’ experiences with symptoms and the effect of LAM on daily life. One FG was conducted with TSC-LAM patients, and six FGs were with S-LAM patients. Each FG lasted approximately 1.5 hours, was conducted in English by a member of the study team (A.B. or J.S.) trained in qualitative data collection, and was digitally recorded and then transcribed verbatim. The study was approved by the Institutional Review Board at National Jewish Health in Denver, Colorado (HS-2774); written informed consent was provided by all participants.

FG data were analyzed in an iterative process involving established qualitative content methods and reflexive team analysis [9-11]. The qualitative data software program ATLAS.ti v.7.0 (Scientific Software Development, GmbH, Berlin) was used for data management and analysis. FG transcripts were read multiple times to achieve immersion, and code categories were then developed using an emergent rather than *a priori* approach [11]. Members of the study team applied the resulting codes to the transcripts, meeting regularly to check new findings, discuss emergent new codes and themes, and assess the preliminary results of the analysis process [12,13].

## Results

Thirty-seven women each participated in one FG (n = 5 for the TSC-LAM FG and n = 5, 5, 6, 6, 5, and 5 for the six S-LAM FGs). Participants’ baseline characteristics are displayed in Table 1. Seven primary symptoms and five common psychological experiences were emergent in the data. Participants identified shortness of breath, low energy, cough, sensations in the chest, difficulty sleeping, gastrointestinal issues, and mild cognitive difficulties as the primary physical symptoms of LAM, while frustration, worry, loss of identity, embarrassment, and defiance were identified as the common psychological experiences (Table 2).

**Table 1:** Baseline characteristics of 37 focus group participants with LAM

	
Age in years	52 (1st quartile - 3rd quartile 47–61; range 34–68)
Female	37 (100%)
Ethnicity*	
Non-Hispanic	34
Hispanic	1
Race*	
Asian	1
Black	0
Other	1
White	33
LAM	
Sporadic	32
TSC	5
LAM duration in years	7.0 (1st quartile - 3rd quartile 2.2-15.2; range 0.4-39.0) N = 33
FEV_1_%	66 (1st quartile - 3rd quartile 39–74; range 2–99) N = 30
Using supplemental oxygen	16 (55%) N = 29
Taking sirolimus	18 (72%) N = 25
Work status**	
Full-time	13
Part-time	10
Disabled	6
Retired	3
Other	2
Education: Bachelor’s degree or more**	26 (76%)

*N = 35; **N = 34; Values are counts (%) or median (1st quartile - 3rd quartile; range).

**Table 2:** Disease-related topics mentioned by focus group participants

**Topic**	**Number of the seven focus groups in which the topic was mentioned**
Symptoms	
Shortness of breath	7
Low energy	7
Cough	7
Sensations in the chest	5
Difficulty sleeping	6
Gastrointestinal issues	4
Mild cognitive difficulties	5
Psychological experiences	
Frustration	7
Worry	7
Loss of identity	7
Embarrassment	6
Defiance	5

### Symptoms

Shortness of breath and low energy were ubiquitous among participants; roughly half of the sample described one or the other as the predominant symptom of LAM.

Participants described shortness of breath with various activities, including showering, washing their hair, walking on level surfaces, walking while carrying even relatively light loads, walking up inclines, and climbing stairs. Walking and talking simultaneously was not possible for many women, because they would become profoundly short of breath. Some recognized increased shortness of breath after eating. As one participant said, “Little by little there is less I can do comfortably [without getting short of breath]”. Because of shortness of breath, participants often found themselves “strategizing every task”, going through mental calculations to determine whether they had the breath or energy to complete them. Tasks or committed daily living activities (e.g., housework) were done “in spurts” and with advanced planning and preparation. One participant described putting items “at the bottom of the stairs to take upstairs [in] one trip instead of three or five that you used to do”. Participants also stressed the need to pace themselves throughout the day; however, many were also frustrated that their slower pace prevented them from “getting as much done” as they had in their “pre-LAM days”. Participants also reported that shortness of breath seemed to vary with weather patterns, becoming more severe and limiting on days with extremes in temperature (high or low), high humidity, or strong wind.

Low energy was referred to in various terms, including “tiredness”, “extreme exhaustion”, “absolutely crushing fatigue” and feeling “wiped”. Although participants tried to “[make] sure [they] have the energy for all of the needs [of the household]”, some described feeling such profound fatigue that they could not meet those needs. In the words of one woman, “forget dinner for the family. I’m done”. Not only was low energy itself an impediment to accomplishing daily tasks, attempts to overcome it were frustratingly time-consuming. One participant noted that “it seems to take up a lot of time just to rest”. Understandably, low energy even affected self-image. As one participant put it, “I don’t like being tired. I like who I used to be”.

Although less commonly mentioned than shortness of breath and low energy, cough was present in many participants. Most indicated that coughing was worse in the morning and frequently occurred when talking. Wheezing was variably present, but not described as particularly prominent or bothersome. Many participants had had one or more episodes of pneumothorax (PTx) throughout the course of their illness. However, at random times, when PTx was determined not to be the cause, these women—as well as many women who had never had a PTx—would experience a nagging heaviness or aching in the chest, sometimes described as a “twinge” in the lungs. Several participants reported the ability to sense and even predict barometric pressure changes based on how their chest felt.

Poor sleep quality, abdominal bloating, and difficulty thinking were also described by some participants. Most desired to sleep more than they were able to. Many also commonly experienced occasional episodes of waking up with a start, feeling like they “forgot to breathe”. Some participants described abdominal distension, bloating and the inability to eat as much as they once did. About the bloating, one participant stated, “…by the end of the day, I’d look like I was three months pregnant”. Finally, some participants also reported mild cognitive difficulties, described variably as “brain fog” or in the context of specific activities, such as “missing words while reading or writing”.

### Psychological experiences

A quote stated by different women in two separate FGs was a thread that wove its way throughout all FGs and encapsulated the psychological experience of living with this disease was “getting stuck with LAM”. For some, “getting stuck is, I think, emotionally with LAM”—not being able to move past the fact that they had been diagnosed with a progressive, incurable disease. For other women, “getting stuck” conveyed their feelings of having been dealt a bad hand.

In the paragraphs that follow, we flesh out the five main psychological themes that emerged, including frustration and resentment, worry and fear, loss of identity, embarrassment and guilt, and finally defiance and resilience.

#### Frustration and resentment

Participants were clearly frustrated by the burdens LAM placed on their lives and the way the disease seemed to make them “…feel like your life is just totally ripped out from under you”. Participants resented how indiscriminately LAM “[took] a lot of experiences from my life”, as one young participant tearfully stated. They resented the intrusion on their ability to live as carefree as they would like, as one participant expressed, “I am a very spontaneous person, and I cannot stand how I have to constantly make these stupid adjustments…and as I get sicker, I’m going to have to make more of them”. Women sensed living with a “pressure to keep up” that was, for many, difficult to deal with: “I feel like I’m on edge all the time. And I just wish I would be allowed to do things at my own pace”.

Many participants’ frustration and resentment left them wondering what they did to deserve LAM; as one participant questioned, “I’ve been a good girl…done what I should do…so, now why am I not able to breathe? I’ve taken care of myself, what’s the deal?” LAM’s omnipresence was inescapable, as one participant sighed, “I’m waiting for the day I don’t think about it [having LAM] just once during the day”.

#### Worry and fear

There was worry about “…what is in store for me in the future… [if LAM progresses and symptoms worsen]”. Many women were trying to manage a household, to be a mother to small children and/or work outside the home, while living with the specter of LAM—and the “…fear that there is something that is going to happen that I can’t manage…” or wondering “what kind of burden I’m going to put on my family [as she ages and LAM progresses]”. Many participants lived with the constant worry that any fleeting sensation could be due to LAM and a harbinger of disease progression. Like other patients with chronic respiratory illness, for participants not using supplemental oxygen, the threat of needing it loomed forebodingly (Question: Do you have fears? “Yeah…being tethered to oxygen all the time [laughing]…more so than death [laughing].”).

#### Loss of identity

Some participants perceived that LAM had taken away their identity (as a worker, an efficacious person, a productive member of society, etc.)—as one woman said “[LAM is] making of my life small, because of the tiredness”. Some women felt like they had lost their independence, including one who said, “I have to rely on my husband to do a lot of things” and another who echoed, “I don’t mind asking for help, but I resent it”. From the combination of dyspnea and fatigue, many women had to dramatically change their lives, and for the active women, this was perceived as a loss of identity: “…my active lifestyle has just taken a back seat to just trying to manage to get through the day”.

#### Embarrassment and guilt

Participants’ physical limitations or their inability to work or their need to use supplemental oxygen (or these in combination) made women feel embarrassed or guilty about “appearing weak” and generally “bad [about] having to say ‘let’s go slower’ because I don’t want to go slower”.

#### Defiance and resilience

Many comments demonstrated that women had moved past the initial shock of being diagnosed with LAM (“Initially [at the time of diagnosis] you go through all these emotions, [and] now you might have an occasional moment, but you don’t get stuck there anymore.”). Several participants described how they had adapted in many ways to life with LAM: physically (“we [she and her husband] still go together and enjoy the things we used to enjoy [going on bike rides], it’s just at a different pace.”); and emotionally (“I decided to be in charge of it [living with LAM]. I put myself back in charge again and said ‘ok’”).

There was a sense of defiance to “just keep living with this [LAM]” no matter what challenges the disease brought; a commitment to “finding something you can do that makes you happy and that you feel like you are contributing”; and to “let go a little bit and let people take care of me”. One participant who used supplemental oxygen described how she was committed to continuing to live her life on her terms, “[women who see me at the gym say] ‘you are such an inspiration! You are here with oxygen’…and I said, ‘I am not an inspiration. I am just stubborn’”.

## Discussion

We recruited 37 women with either TSC- or S-LAM to participate in one of six FGs to gain an improved understanding of the symptoms and other effects of living with the disease. This is the first such study of patients with LAM. In a disease as rare as LAM, 37 is an impressive number of subjects—one that we could have never achieved without the willingness of the LAM community to allow us to conduct our study at LAMposium.

Not surprisingly, exertional dyspnea was perceived as a prominent symptom—it was present with activities ranging from low-energy-demand-activities, like washing hair, to high-energy-demand-activities, like walking up inclines. In the National Heart Lung and Blood Institute’s (NHLBI) study of 203 women with LAM, dyspnea was the most prominent symptom, present in over 70% of subjects at baseline [7]. Wheezing was reported by 46% and cough by 31% of participants in that study. In our cohort, we were impressed by the prominence of fatigue, both in terms of how frequently it was mentioned across FGs and how severely impairing it was for so many women. We suspect fatigue was not asked about specifically in the NHLBI study. We were also struck by the perceived influence of weather on the severity of shortness of breath and other chest symptoms in our FG participants. Another symptom mentioned by several women—but one that we have not had LAM patients volunteer to us in clinic—was abdominal distension and feeling bloated. None had chyloperitoneum or other intra-abdominal abnormalities to account for this symptom. The fact that each of the seven symptoms and five psychological experiences was mentioned in more than half (at least four) of the focus groups (and half the topics were mentioned in all seven focus groups) supports the validity of these themes as capturing the perceptions of LAM patients.

The paucity of published data makes placing the results of our study in the context of what is known about the psychological effects of living with LAM difficult. In the NHLBI study, scores from the Mental Component Summary of the SF-36 (a summary measure that includes items which ask about various aspects of mental health including mood), were no different from normative values from the US adult general population [7]. However, questionnaires are unable to fully capture the richness of data obtained from FGs. Among our participants, psychological effects were varied and hinged, at least partially, on stage of illness. For most women, being handed a diagnosis of a chronic, progressive disease was devastating, placing women at risk for getting “stuck” in an emotional valley. Once the dread of being diagnosed had passed, women faced the daily frustrations of living with LAM. This included dealing with symptoms and being forced to adjust to LAM-imposed lifestyle modifications, including doing things at a slower pace, strategizing and rationing energy to make it through the day. These, and the other psychological experiences we observed, including fear and worry, loss of identity, embarrassment and guilt are common to patients with other chronic, debilitating lung diseases [14]. For example, among patients with pulmonary hypertension (PH, another disease that predominantly affects females), McDonough and her colleagues identified two main themes to describe the experience of living with PH—“holding back” and “redefining life” [15]. Holding back included components and subthemes like shortness of breath (the predominant symptom observed), fear of the effects of physical overexertion, anticipation of disease progression, and the inability to live with spontaneity; while “redefining life” was driven by some being forced to use supplemental oxygen, living with uncertainty and adapting to a new lifestyle within the confines of PH.

Future research should focus on whether and how the burdens of LAM can be lessened. For example, do LAM patients derive benefits in functional capacity, symptom severity and quality of life from traditional pulmonary rehabilitation (PR) programs? We suspect they do, but to our knowledge, this has not been systematically examined. Investigators could assess each component of PR to develop new, or revise existing, practices to maximize their benefit for LAM patients. It was abundantly clear to us that LAM patients (and their families) enjoyed the camaraderie and educational environment of LAMposium. More than that, LAMposium united, unified and empowered LAM patients against the disease. Through this meeting and several other efforts, the LAM Foundation has become a model for patient advocacy, networking and research for rare diseases.

As is common in qualitative research, this study is potentially limited by its relatively small sample size. However, for a rare disease like LAM, 37 subjects is not a trivial amount. Furthermore, like other qualitative studies, the goal here was to seek depth rather than breadth, and we believe we achieved our goal. It is possible that the women who participated in these FGs are not representative of others living with LAM. While we sought to mitigate this possibility by conducting focus groups at the LAMposium meeting, which draws a diverse group of patients from across the nation, it is nonetheless difficult to determine generalizability to the larger LAM population, including patients who were unable or unwilling to travel. We do not have data on how many women actively participated in support groups; one could reasonably expect that those who did could have a different emotional experience of living with LAM. Reflecting the new landscape of LAM, the majority of participants were taking sirolimus. This could have influenced experiences in multiple ways: drug side effects could be responsible for certain physical symptoms and/or the hope that—for the first time—having an effective therapy for LAM might have positively affected emotional well-being. Finally, the women with TSC may have been affected by non-LAM aspects of their disease; given that our focus was on LAM (and not other manifestations of TSC, like the dermatological or neurological), we consciously chose not to identify those aspects or probe to discern their impact on the TSC-LAM patients’ daily lives.

In summary, we conducted FGs with 37 women who have either TSC- or S-LAM and captured a rich cache of data on their physical and psychological experiences of living with this disease. The most imposing symptoms were shortness of breath and fatigue, and the psychological experience was dominated by frustration and worry. Additional research is needed to determine whether and how the burdens of this disease can be mitigated.

## Competing interests

The authors declare that they have no competing interests.

## Authors’ contributions

AB, KA, JS: Study conceptualization; AB, KF, JD, JS: Data collection; KA, JS: Data analysis; AB, KA, KF, JD, JS: manuscript preparation and editing. All authors read and approved the final manuscript.
